# Comparing executive functions profiles in anorexia nervosa and autism spectrum disorder in adolescence

**DOI:** 10.1002/erv.2904

**Published:** 2022-04-11

**Authors:** Clément Ghiotto, Catarina Silva, Isabelle Charvin, Paola Atzori, Marion Givaudan, David Da Fonseca, Flora Bat‐Pitault

**Affiliations:** ^1^ Child and Adolescent Psychiatry Unit Salvator University Hospital Public Assistance‐Marseille Hospitals Aix‐Marseille University Marseille France; ^2^ Institute of Neuroscience Timone CNRS Aix‐Marseille University Marseille France

**Keywords:** adolescence, anorexia nervosa, autism spectrum disorder, executive functions, set‐shifting

## Abstract

**Objective:**

Executive functions (EFs) inefficiencies in anorexia nervosa (AN), especially in set‐shifting and central coherence, suggest a link between AN and autism spectrum disorders (ASDs). This study aimed at comparing EF profiles in AN and ASD, and investigating clinical variables associated with the identified EF difficulties.

**Method:**

One hundred and sixty‐two adolescents with AN or ASD completed self‐report questionnaires assessing depression, anxiety and autism symptoms. Parents completed the behaviour rating of executive functions parent‐form (BRIEF‐P). Besides comparing EFs in AN and ASD, we also analysed clinical variables scoring below and above the mean age score across the all sample. We additionally examined the relationship between clinical variables and the BRIEF‐P indexes in AN.

**Results:**

Participants with ASD had greater EF difficulties than participants with AN on all BRIEF‐P scales. In the whole sample, higher autistic features were related to poorer EF. In AN, lower body mass index and particularly higher autism‐spectrum quotient (BRI: Beta = 0.55; *p* < 0.001 and GEC: Beta = 0.50; *p* < 0.001) were most strongly associated with poorer EF.

**Conclusion:**

Although participants with ASD showed greater difficulties, autistic traits were related to alter EFs in AN. Exploring further this dimension can undeniably allow better adaptive cognitive remediation programs.

## Highlights

- Participants with autism spectrum disorder (ASD) had greater executive function (EF) difficulties than participants with anorexia nervosa (AN) on all behaviour rating of executive functions parent‐form scales
- In AN, lower body mass index and particularly higher autism‐spectrum quotient were most strongly associated with poorer EF
- Our study highlights the importance of autistic features in the alteration of EF in AN

**Abbreviations**
ADI: autism diagnostic interview
ADOS: autism diagnostic observation schedule
AN: anorexia nervosa
ANBP: anorexia nervosa bingeing/purging subtype
ANR: anorexia nervosa restrictive subtype
AQ: autism spectrum quotient
ASD: autism spectrum disorders
BMI: body mass index
BRI: behavioural regulation index
BRIEF‐P: behaviour rating of executive functions parents‐form
CDI: child depression inventory
CRT: cognitive remediation therapy
D‐KEFS: delis‐kaplan executive function system
DSM‐5: diagnostic and statistical manual 5th edition
EDI‐2: eating disorder inventory‐2
EF: executive functions
GEC: global executive composite
IQ: intelligence quotient
K‐SADS: kiddie schedule for affective disorder and schizophrenia
MI: metacognition index
ROCFT: rey osterrieth complex figure test
STAI‐Y: state‐trait anxiety inventory form
TBI: traumatic brain injury
TMT: trail making test
WCST: wisconsin card sorting test
WISC‐IV: wechsler intelligence scale for children 4th edition

## 1 INTRODUCTION AND AIMS

Anorexia nervosa (AN) is an eating disorder characterised by persistent restriction of calorie intake, a fear of gaining weight and a disturbance in body perception (Kaufman et al., 2000). It is a major public health concern affecting people world‐wide. In France, for example, an epidemiologic study showed that AN affects 0.5% of adolescent girls (Godart et al., 2013). Other studies have found that AN has a life‐long prevalence varying from 1% to 4% for cohort studies, and 1% for two‐phase studies (Smink et al., 2012). With limited effectiveness of current treatment options, management remains difficult with a 46.9% recovery rate, 33.5% of improvement, 20.8% of chronicisation, and 5% of mortality (Steinhausen, 2009). The standardised mortality ratio is 5.86 in AN, 20% of which are deaths by suicide (Arcelus et al., 2011). Moreover, even after the end of treatment, a lower body mass index (BMI) and a higher incidence of eating‐related symptomatology are observed compared to healthy controls (Tomba et al., 2019). In addition, the disorder seems to have a significant impact on the quality of life (Ágh et al., 2016).

In light of these outcomes, research has focussed on cognitive functioning in AN, and on executive functions (EF) in particular. This increased interest underlines the goal of developing better and more specific treatment strategies. To date, few studies have found differences in cognitive functioning between children and adolescents with AN and control participants. Kjaersdam Télleus et al. (2015) for example, found no differences in the full scale intelligence quotient (IQ) in a sample of 94 children and adolescents with AN compared to controls. However, results showed that adolescents with AN had a more heterogeneous cognitive profile compared to controls, performing significantly better in verbal (Verbal IQ and Verbal Comprehension Index) than in nonverbal (Perceptual Organization Index and Performance IQ) tests. This study also pointed out a more detailed‐oriented approach in participants with AN relative to controls, with a better performance in the Rey Osterrieth complex figure test (ROCFT). This is in line with studies showing poorer short‐term verbal memory in AN (Lao‐Kaim et al., 2014), and with the hypothesis that weak central coherence may be a possible endophenotype for AN (Lang, 2014). Although interesting, the data on the topic is still conflicting. Another example is Bentz's et al. study (Bentz et al., 2017), in which young females with first‐episode AN and those recovered from AN performed tests assessing working memory, processing speed, sustained attention, verbal memory and verbal abstraction, at comparable levels as control participants.

Among cognitive functions, EF can be defined as a set of mental processes that, in light of new information, allow establishing goals, generating strategies or sequencing complex actions to achieve those goals, while successfully monitoring behaviour. The EF are of particular interest because of their interplay with the regulation of behaviour and emotion (Godefroy et al., 2008). Several studies in AN, including a meta‐analysis by Hirst et al. (2017), report difficulties in set‐shifting and central coherence in acute as well as recovered adult patients. However, it is not clear whether age and duration of illness play a role, as these difficulties are more frequent in adults than in adolescents (Hirst et al., 2017). This is still a question open to debate. Indeed, some studies comparing children and adolescents with AN to matched controls found no differences in set‐shifting, as assessed by the Trail Making Test and the Cambridge Neuropsychological Test Automated Battery (Kjaersdam Telléus et al., 2015), or the Delis‐Kaplan Executive Function System (D‐KEFS (Bentz et al., 2017);). This absence of between‐group differences was reported by other studies, either using neuropsychological tests (e.g., verbal fluency, Wisconsin card sorting test (WCST), Brixton's Task (Calderoni et al., 2013; Fitzpatrick et al., 2012), or more ecological approaches, with the Behaviour Rating of Executive Functions (BRIEF; Dahlgren et al., 2014; Herbrich et al., 2018)).

These findings showing no EF impairments in participants with AN relative to controls seems nevertheless to contradict the current literature comparing AN subtypes. While this issue places EF assessment in AN at the core of research studies, results remain unclear. Herbrich et al. (2018) found more EF difficulties, in particular in set‐shifting in the binging/purging AN subtype (ANBP) than in the restrictive subtype (ANR), as assessed by the self‐report form of the BRIEF, whereas Galimberti et al. (2013) found no between‐group differences in set‐shifting as assessed by the WCST. This indeed leads us to the question of the adequacy of assessment tools for EF. The evaluation of EF by neuropsychological tests is evolving with the emergence of progressively more ecological tests. Historical neuropsychological tests such as the WCST, the Go/No‐Go test or the ROCFT were initially designed to assess EF for diagnostic purposes in adult patients with brain injuries (Chaytor & Schmitter‐Edgecombe, 2003). According to Chaytor & Schmitter‐Edgecombe (2003), there is a moderate association between neuropsychological tests and daily cognitive functioning, while verisimilitude tests (simulating a situation closer to daily life than conventional tests, i.e., more ecological) showed a stronger preliminary association. Relatedly, Stedal and Dahlgren (2015) found that performance‐based scores (using the neuropsychological test battery 'Ravello Profile’) did not substantially correlate with self‐report assessment (using the BRIEF‐SP) in a group of 20 female adolescents with AN. Spitoni et al. (2018) also found that while classical tests detected few differences in EF between patients and healthy controls, ecological tests such as the Zoo Map test were more sensitive, consistently and systematically identifying significant altered EF (e.g., speed of completion) in a group of female patients with AN. This is a critical topic requiring additional research.

Nonetheless, set‐shifting and central coherence difficulties have been reported in adults with AN, and raise the question of a possible link between AN and autism spectrum disorder (ASD). Indeed, and similar to the EF deficits found in AN (Hirst et al., 2017) a meta‐analysis by Demetriou et al. (2018) reports that the EF deficits found in patients with ASD include areas such as working memory, inhibition, verbal fluency, mental flexibility and concept formation, with moderate to large effect sizes. In fact, this possible link between AN and ASD is not new. As early as 1983, Gillberg (1983) raised this question, highlighting repetitive and ritualistic behaviours around meals as well as difficulties in social relationships in patients with AN. Dell'Osso et al. (2016) further provide arguments in favour of this hypothesis: an over‐representation of the diagnosis of ASD in patients suffering from AN, a ritualisation and rigidity which is however crystallised around food and weight, difficulties with set‐shifting tests, an information processing style detail‐oriented signalling difficulties in central coherence, as well as difficulties in certain theory of mind tests. Furthermore, Westwood et al. (2017) found that among 99 adolescents and adults with AN, those with more autistic traits, as assessed by the autism diagnostic observation schedule 2 (ADOS‐2), displayed more set‐shifting difficulties at the WCST.

Thus, in this study we aimed at investigating the EF profile of adolescents with AN compared to that of adolescents with ASD using the behaviour rating of executive functions parent form (BRIEF‐P). As mentioned earlier, literature on EF deficits suggests a potential link between AN and ASD (Dandil et al., 2020; Lepannen et al., 2018). In the case of ASD, cognitive remediation therapy (CRT) may be an effective intervention on such deficits (Dandil et al., 2020). It may be possible that a deeper understanding of the EF profile in AN, especially in those with higher autistic traits, may drive as well the development of specific CRT interventions targeting EF alteration in AN (Giombini et al., 2022; Lepannen et al., 2018; Tchanturia et al., 2014, 2016). To do so, we chose an ecological approach by means of the BRIEF scales. Previous studies have shown that assessing EF using a performance‐based approach found no executive deficits in AN, while those using the Eating Disorder Inventory showed a strong feeling of self‐ineffectiveness and remain a key feature of the disorder, pointing towards important daily life difficulties. The ecological approach of cognition assessment, that is, one more closely related to everyday behaviour, has been initially developed in the field of traumatic brain injury (TBI). A study of Mangeot et al. (2002), focussing on EF in particular, found that in children with TBI, performance‐based scores demonstrated modest associations with the BRIEF‐P ratings. Conversely, the BRIEF‐P ratings were strongly associated with measures of emotional and behavioural adjustment and adaptive behaviour. In short, the BRIEF may be more sensitive than conventional neuropsychological tests for detecting daily life difficulties in AN, especially in adolescence.

Considering the consistent findings of EF deficits in adolescents with ASD as opposed to those with mixed data in adolescents with AN, we hypothesised that participants with ASD would present more EF alterations than participants with AN. In line with Herbrich et al. (2018), we further hypothesised that, among participants with AN, the ANBP subtype would be associated with more difficulties than the ANR subtype. We additionally investigated the clinical variables related to altered EF in a transdiagnostic manner in the whole sample. This further lead us to a focus in particular on the clinical variables related to EF variations in AN, with the hypothesis that autistic traits may be associated with their EF difficulties, in line with Westwood et al. (2017).

## 2 METHOD

### 2.1 Participants

We analysed data from 162 participants, aged from 8 to 18 years old, with AN or ASD. These patients were admitted for evaluation and/or care in the child psychiatry department of the Salvator University Hospital in Marseille between 2018 and 2020, on a full‐time in‐ or outpatient basis. Patients presenting an intellectual disability, as assessed by the Wechsler Intelligence Scale for Children 4th Edition (WISC‐IV), were excluded from the study, as well as those patients whose parents did not complete the BRIEF‐P at the time of assessment.

### 2.2 Procedures

Participants' assessments were conducted on a 2‐day outpatient basis for patients with AN or ASD, and resulted in the referral to the local child psychiatry network or the initiation of management for patients in day hospital or full‐time inpatient care. Assessment of participants with AN were multidisciplinary (psychiatrist, psychologist, dietitian), and used the semi‐structured interview kiddie schedule for affective disorder and schizophrenia (K‐SADS; Kaufman et al., 2000), which provides a diagnosis of AN according to the criteria of the Diagnostic and Statistical Manual, 5^th^ Edition (DSM‐5; American Psychiatric Asso, 2013). BMI and duration of illness were also collected for the group with AN. Assessment of participants with ASD were conducted using semi‐structured interviews: the autism diagnostic observation schedule (ADOS; Lord et al., 1989) for adolescents and the autism diagnostic interview (ADI; Le Couteur et al., 1989) for parents. Adolescents with AN or ASD then completed several self‐administered questionnaires, including the child depression inventory (CDI, Kovacs, 1985; Saint‐Laurent, 1990) and the state‐trait anxiety inventory form Y (STAI‐Y, Spielberger, 1983; Gauthier & Bouchard, 1993), and the autism‐spectrum quotient (AQ, Baron‐Cohen et al., 2006), while their parents completed the BRIEF‐P (Gioia et al., 2000). The patients with AN additionally fill the eating disorder inventory 2 (EDI‐2 (Criquillion‐Doublet et al., 1995).

The BRIEF‐P is a standardized rating scale assessing EF in children and adolescents aged from 5 to 18 years old. The scale is completed by a parent (ideally both). It includes normative data (*T*‐scores from 0 to 100) that can be transformed in standardized *z*‐scores (a *z*‐score > 1.5 SD, equivalent to a *T*‐score > 65, is considered clinically significant, thereby reflecting higher levels of problems or difficulties). The BRIEF‐P is composed of 86 items targeting EF‐related behaviours rated on a 3‐points Likert scale (from ‘never’ to ‘often’), divided into 8 clinical scales and 2 validity scales. The ‘inhibition’, ‘shift’ and ‘emotional control’ scales compose the behavioural regulation index (BRI), and the ‘initiate’, ‘working memory’, ‘planning/organization’, ‘organization of materials’, ‘monitor’ scales compose the metacognition index (MI). The BRI and MI can be combined to obtain the global executive composite (GEC). The BRIEF‐P's test review (Gioia et al., 2000) revealed that the scale has a good reliability, with a Cronbach α of 0.80–0.94 and a correlation coefficient *r* of 0.81. We chose this scale over performance‐based testing and the self‐report version of the BRIEF for several reasons. Neuropsychological tests are highly structured, providing guidance leading to optimal executive performance, which may in fact reduce the actual demands on the EF and thus reduce their actual implication on test performance, as well as their ecological validity. By contrast, the BRIEF allows to assessing EF in a less artificial manner, capturing actual manifestations of executive functioning and their impact in patients' everyday lives (Gioia et al., 2008). This is particularly relevant in adolescents with AN for whom no major executive deficit has been demonstrated, as measured by traditional neuropsychological testing. Although other aspects than EF may impact the BRIEF scores, these remain a good reflection of daily life difficulties that adolescents with AN may encounter and that need to be addressed. Secondly, although parent‐report may be biased, Herbrich et al. (2018) found that both the BRIEF‐P and the BRIEF‐self report showed only low to moderate associations with the performance‐based tests. Moreover, while patients reported more EF difficulties than their parents, both scores fell within the normal range (Herbrich et al., 2019). In the context of establishing a diagnosis taking place over 2 half‐days, the acceptability for certain patients with a very low BMI at the time of assessment, and considering the number of self‐report questionnaires, we chose the BRIEF‐Parent form over the self‐report form.

As previously mentioned, the study included other self‐report questionnaires: The EDI‐2 is a 91‐item questionnaire assessing eating behaviour in 11 dimensions (asceticism, bulimia, interoceptive awareness, impulse regulation, drive for thinness, ineffectiveness, body dissatisfaction, social insecurity, interpersonal distrust, perfectionism and maturity fears). Statements are scored on a 6‐points Likert scale (0—‘never’, 1—‘rarely’, 2—‘sometimes’, 3—‘often’, 4—‘usually’ and 5—‘very much so’). Each item is scored by transforming scores ranging from 0 to 3 rather than 0–5: a score from 1 to 3 is assigned to ‘symptomatic’ response (always = 3, usually = 2 and often = 1), and 0 is assigned to the three ‘asymptomatic’ responses (sometimes, rarely and never). Higher scores indicate a higher eating‐related symptoms severity (Criquillion‐Doublet et al., 1995). The CDI assesses depressive symptoms in 27 statements, each one consisting of three statements that are graded in severity and are assigned numerical values from 0 to 2.27. Scores higher than 15 indicate a major depressive episode (Kovacs, 1985; Saint‐Laurent, 1990). The STAI‐Y assesses anxiety symptoms *in the present moment* (form STAI‐Y‐1, 20 items) and in *general* (form STAI‐Y‐2, 20 items). Each statement is scored on a 4‐points Likert scale (from 1—‘not at all’ to 4—‘very much so’). Scores range from 20 to 80. Higher scores indicate more anxiety (Spielberger, 1983; Gauthier & Bouchard, 1993). The AQ screens for autistic traits in adolescents without intellectual disability. It is composed of 50 items assessing 5 areas of interest: social skill, attention switching, attention to detail, communication, and imagination. Items are scored on a 6‐points Likert scale ranging from 0 to 1 (from ‘disagree’ to ‘agree’). Higher scores indicate more autistic traits (Baron‐Cohen et al., 2006).

All adolescents included in this study were volunteers. The experiment adhered to the Declaration of Helsinki and experimental procedures were approved by the Ethics Committee of Aix‐Marseille University (N/Ref: 2020‐12‐03‐003) and the local general data protection regulation (Ref: RGPD/AP‐HM 2020‐138). Participants and/or parental informed consent were obtained from all participants at the time of testing.

### 2.3 Statistics

The statistical analyses were conducted using SPSS 20.0 software. Descriptive analyses were performed using means and standard deviations for continuous variables, while categorical variables were presented in number of participants and frequencies. A non‐parametric approach was used to account for the non‐normality of the frequency of certain variables. First, Mann–Whitney and chi‐square *U*‐tests were conducted to compare EF and clinical characteristics of participants with AN and ASD. Bonferroni corrections were used in the multiple comparisons for the BRIEF sub‐dimensions. We then used Kruskall–Wallis and Dunn's post‐hoc tests to compare the scores of the BRIEF Indexes between the three diagnostic groups (ANBP, ANR, and ASD). Univariate Mann–Whitney and chi‐square *U*‐test analyses were used across the entire sample to compare, for each BRIEF Index (BRI, MI, and GEC), the clinical variables in the group of participants scoring below the mean age score (Z‐score < 0) and those in the group scoring above the mean age score (Z‐score > 0). Variables significantly associated with the BRIEF Indexes studied at *p* < 0.10 in the univariate analyses were then included in the multivariate analysis. This was carried out with logistic regressions that allows the calculation of odds ratios and indicates the associations that remain significant at *p* < 0.05 with the BRIEF Indexes studied after simultaneous adjustment of the other variables. Finally, linear regressions were used to study the relationships between the different clinical variables with the three BRIEF Indexes (BRI, MI and GEC) on the participants with AN, as well as the relative weight of these variables in the evolution of these Indexes. All statistical tests were performed bilaterally with an alpha of 5% and *p*‐values below 0.05 were considered significant.

## 3 RESULTS

Sample characteristics indicated that from the 162 participants included, there were 20 boys (12.3%) and 142 girls (87.7%) aged 8–18 years old, with an average age of 14.42 ± 2.18 years. The AN group was composed of 72 participants (71 girls or 98.6%) aged 8–18 years old, mean age 14.42 ± 2.50, mean BMI 15.46 ± 2.33, with a mean duration of illness of 9.98 months ± 4.51. The AN group consisted of 59 patients with ANR (81.9%) and 13 patients with ANBP (18.1%). The ASD group included 90 participants (71 males or 78.9%) aged 10–18 years, mean age 14.41 ± 1.91 years.

The results of the comparisons of EF and clinical characteristics between the AN and ASD groups are presented in the Table 1. Significantly greater difficulties were found in the ASD group compared to the AN group for all eight BRIEF‐P scales and all three Indexes (BRI, MI, and GEC), even after Bonferroni correction (*p* < 0.05/11, i.e., at *p* < 0.004). Analyses also showed significantly higher levels of anxiety in the AN group after Bonferroni correction.

**TABLE 1:** Executive functions and clinical characteristics of the sample in relation to the psychiatric disorder (AN or ASD)

	AN	ASD
** **	*N* = 72 Mean ± Standard deviation or *N* (%)	*N* = 90 Mean ± Standard deviation or *N* (%)
Sex^a^**		
Girl	71 (98.6)	19 (21.1)
Boy	1 (1.4)	71 (78.9)
Age^b^	14.43 ± 2.50	14.41 ± 1.91
CDI total score^b^	16.71 ± 9.65	16.39 ± 8.65
STAI form Y‐1^b^*	48.29 ± 15.07	39.49 ± 15.04
STAI form Y‐2^b^	51.71 ± 14.07	49.85 ± 14.12
BRIEF^b^		
Inhibition**	0.48 ± 1.48	1.92 ± 1.58
Shift**	1.25 ± 1.60	3.33 ± 1.48
Initiate**	0.66 ± 1.55	2.29 ± 1.15
Emotional control**	1.08 ± 1.58	1.91 ± 1.56
Working memory**	0.06 ± 1.23	2.10 ± 1.39
Planning/Organization**	0.01 ± 1.26	2.13 ± 1.25
Organization of materials**	0.04 ± 1.33	1.02 ± 1.14
Monitor**	0.31 ± 1.38	1.80 ± 0.95
BRI**	1.11 ± 1.69	2.74 ± 1.48
MI**	0.23 ± 1.46	2.27 ± 1.16
GEC**	0.59 ± 1.55	2.64 ± 1.24

*Note*: ٭*p* < 0.05 compared to ASD group subjects; **p < 0.001 compared to ASD group subjects.

Abbreviations: BRI, behavioural regulation index; BRIEF, behaviour rating of executive functions; CDI, child depression inventory; GEC, global executive composite; MI, metacognition index; STAI, state trait anxiety inventory.

^a^Data presented as number of subjects (%).

^b^Data presented as mean ± standard deviation.

**FIGURE 1:**
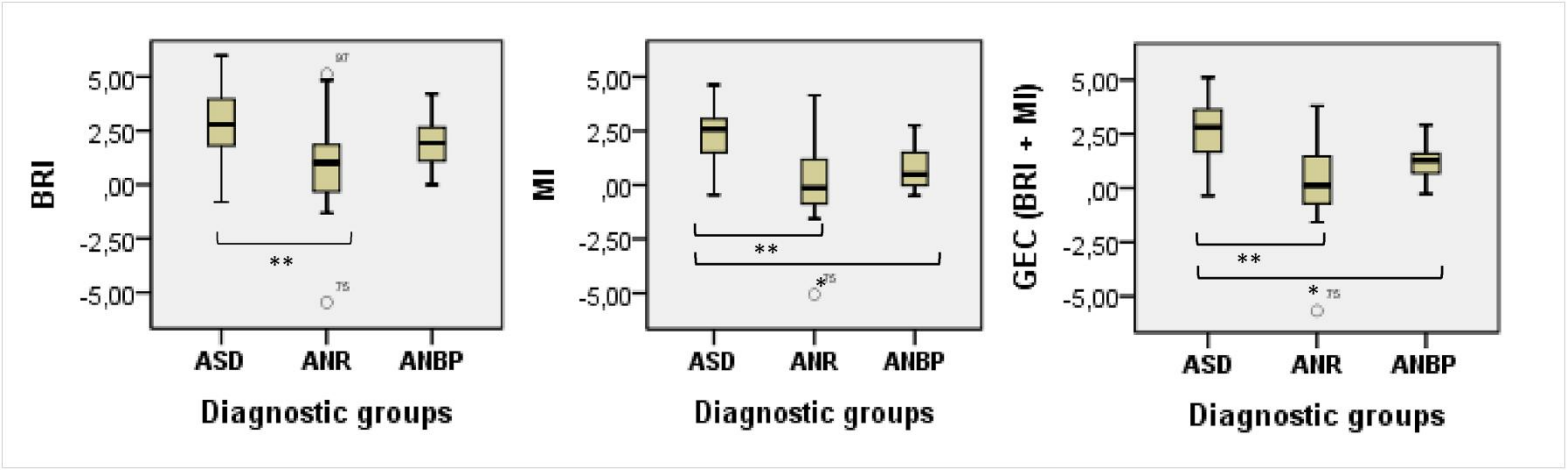
Average behaviour rating of executive functions parent‐form indexes by diagnostic group (anorexia nervosa restrictive, autism spectrum disorder, and anorexia nervosa binging/purging). *p < 0.05. **p < 0.001. ANB/P, anorexia nervosa binging/purging; ANR, anorexia nervosa restrictive; ASD, autism spectrum disorder; BRI, behavioural regulation index; GEC, global executive composite; MI, metacognition index

A closer comparison between AN subtypes and the ASD group showed significant differences between the ASD, ANR, and ANBP groups for the three BRIEF‐P Indexes: BRI (*χ* ^2^ [2] = 37.44; *p* < 0.001), MI (*χ* ^2^ [2] = 64.24; *p* < 0.001), and GEC (*χ* ^2^ [2] = 60.36; *p* < 0.001). Significant differences between the three diagnostic groups, for each BRIEF‐P index, are presented in Figure 1. Results show in particular intermediate executive difficulties in the ANBP group compared to the ASD and ANR groups.

Analyses on the clinical variables in relation to variations in the different BRIEF‐P Indexes are presented in Table 2. For the BRI, which was found to be significantly impaired in the entire sample (2.02 ± 1.77), and to be significantly related to depressive and anxiety features, as well as to ASD diagnosis. After adjustment for other clinical variables, the diagnosis of ASD remained significantly stronger (odd ratio = 28.12) in relation to the BRI. Similar results are found for the MI and GEC.

**TABLE 2:** Clinical variables of the adolescent sample in relation to BRIEF dimensions

		BRI		
	Group Z‐score < 0	Group Z‐score > 0	Odd‐ratio (95% CI)^a,b^	Odd‐ratio(95% CI)^a,c^
** **	*N* = 39	*N* = 139		
** **	Mean ± SD	Mean ± SD		
CDI total score	10.39 ± 7.07	17.59 ± 9.00	1.13 (1.05‐1.21)**	1.08 (0.96–1.22)
STAI‐Y 1	38.39 ± 13.30	46.39 ± 15.73	1.04 (1.00–1.07)	1.04 (0.97–1.11)
STAI‐Y 2	41.00 ± 11.99	53.09 ± 13.59	1.07 (1.03‐1.12)*	0.97 (0.88–1.06)
Diagnosis	*N* (%)	*N* (%)		
AN	20 (87.0%)	52 (37.4%)	1	1
ASD	3 (13.0%)	87 (62.6%)	29.00 (8.39‐100.23)**	**28.12 (5.41**–146.17)**

		**IM**		
	*N* = 39	*N* = 123		
	Mean ± SD	Mean ± SD		
CDI total score	10.39 ± 7.07	17.59 ± 9.00	**1.13 (1.05**–1.21)**	1.06 (0.89–1.25)
STAI‐Y 1	38.39 ± 13.30	46.39 ± 15.73	1.04 (1.00–1.07)	0.98 (0.91–1.06)
STAI‐Y 2	41.00 ± 11.99	53.09 ± 13.59	**1.07 (1.03**–1.12)*	1.07 (0.95–1.22)
Diagnosis	*N* (%)	*N* (%)		
AN	20 (87.0%)	52 (37.4%)	1	1
ASD	3 (13.0%)	87 (62.6%)	11.15 (3.16‐39.36)**	18.11 (1.98‐165.49)*

		**GEC**		
	*N* = 30	*N* = 132		
	Mean ± SD	Mean ± SD		
CDI total score	12.27 ± 8.38	17.54 ± 8.99	**1.08 (1.02**–1.14)*	1.09 (0.95–1.24)
STAI‐Y 1	42.19 ± 14.93	45.95 ± 15.77	1.02 (0.99–1.05)	0.99 (0.93–1.06)
STAI‐Y 2	45.54 ± 13.53	52.82 ± 13.82	**1.04 (1.00**–1.08)*	1.01 (0.92–1.12)
Diagnosis	*N* (%)	*N* (%)		
AN	27 (90.0%)	45 (34.1%)	1	1
ASD	3 (10%)	87 (65.9%)	**17.40 (5.00**–60.48)**	11.58 (2.31–58.10)*

*Note*: * *p* < 0.05. ** *p* < 0.001.

Abbreviations: AN, anorexia nervosa; ASD, autism spectrum disorder; BRI, behavioural regulation index; CDI, child depression inventory; GEC, global executive composite; MI, metacognition index; STAI‐Y, state trait anxiety inventory form Y.

^a^An odds ratio of 1 indicates the reference group. Values shown in bold are significant. The z‐score group < 0 for each BRIEF dimension is the reference group.

^b^Odd ratio calculated by logistic regression for each variable.

^c^Adjusted simultaneously for diagnosis and psychometric data (CDI total score, STAI‐Y 1 and STAI‐Y 2).

Finally, in the AN group, among the six variables (STAI‐Y, CDI, AQ, EDI‐2, BMI, Duration of Illness) studied by the linear regression analyses, the only variables found to be associated with the variation in the three Indexes of the BRIEF‐P were the BMI and the AQ. Changes in the BMI were associated with the largest changes in the MI (Beta = 0.48; *p* < 0.001) while changes in autistic traits, as assessed by the AQ, were associated with the largest changes in the BRI (Beta = 0.55; *p* < 0.001) and in the GEC (Beta = 0.50; *p* < 0.001).

## 4 DISCUSSION

Consistent with the literature, our study does not highlight significant differences in executive functioning between the AN group and the BRIEF's normative data (Timko et al., 2021). Although our study did not include an actual control group, available studies support the good validity of the BRIEF's standard norms, even independent of geographic stratification (Roth et al., 2015). This absence of difference has been reported by other studies, either using traditional neuropsychological tests such as verbal fluency, the WCST, Brixton's Task, and the D‐KEFS and the NEPSY‐II (Bentz et al., 2017; Calderoni et al., 2013; Fitzpatrick et al., 2012; Hirst et al., 2017; Kjaersdam Telléus et al., 2015), but also by those using the BRIEF (Dahlgren et al., 2014; Herbrich et al., 2018). However, a meta‐analyse failed to show the same finding in adolescents with AN, mainly due to the small number of studies in this age group, as well as to the use of mixed adolescent and adult populations (Hirst et al., 2017). One of the main interests of our study is the use of the BRIEF‐P, a more ecological assessment of EF executive functions in everyday life, avoiding the limitations related to neuropsychological tests (e.g., 35). Moreover, our results show significant alterations in the ASD group. This is in agreement with Gardiner and Iarocci (Gardiner & Iarocci, 2018), who show alterations on 5 of the 8 BRIEF scales and on the three BRIEF Indexes in a sample of 126 children and adolescents with ASD, as well as with Zandt et al. (2009), who found alterations in the BRI, MI and the GEC.

Second, and in view of the heterogeneity of studies in the literature, another main interest of our study lies in the fact that it is one of the few directly comparing large sample well‐characterised adolescents with ASD and with AN, using the BRIEF‐P. This should undeniably help a better characterisation of EF in AN, and disentangle what the clinical applications and interventions on ASD may bring to novel treatments options in AN. As hypothesised, present findings showed a significantly different EF profile between adolescents with ASD and AN, in particular in terms of set‐shifting, with significantly greater difficulties in the ASD group. These results are in agreement with a recent study by Timko et al. (2021) which found different executive performance in female adolescents with ASD and in female adolescents with AN using the BRIEF. Present results partially contrast with those reported by Westwood's et al. (2016) meta‐analyses, who's meta‐regression failed to find a significant influence of the AN or the ASD diagnosis on set‐shifting. However, this meta‐analysis focussed on set‐shifting as assessed by a performance‐based test, the WCST. Along the same lines Kerr‐Gaffney et al. (2021) found that the quality of social response in females with AN was significantly better compare to those with ASD. Indeed, Lang (2014), showed that central coherence, classically altered in ASD, is also altered in eating disorders and particularly in AN. In the current study, analyses directly comparing these two groups showed that anxiety was significantly higher in the AN group, but that does not explain the cognitive differences found, as the scores of the ASD group keep reflecting more impairment.

According to our hypothesis, our results may show an intermediate performance profile of the ANBP group. This is consistent with the results of Herbrich et al. (2018). Using the self‐report form of the BRIEF, they found significant differences between ANR and ANBP on the GEC and the BRI but not on the MI, with greater difficulties for the ANBP group. Importantly, these results are reported for a sample similar to ours in terms of age, BMI and duration of illness. Unfortunately very few studies exist preventing us from directly contrast the present findings. There are however a few studies showing similar profiles between ANR and ANBP on set‐shifting as measured by the WSCT (Galimberti et al., 2013; Van Autreve et al., 2013) and by the trail‐making‐test (TMT, Van Autreve et al., 2013). Other studies focussing on inhibition show conflicting findings, with some reporting greater motor impulsivity in adults with ANBP (Rosval et al., 2006; Wu et al., 2013), and others showing no brain activation differences between adolescents with ANR and ANBP in a go/no‐go task (Lock et al., 2011). It is possible that higher levels of impulsivity, a hallmark feature of the ANBP subtype, may alter their daily life functioning. However, literature on this topic is to date limited and inconsistent to draw conclusions.

In addition, our results showed a significant association between anxiety‐depressive features and changes in the BRIEF‐P scores. This is consistent with Dahlgren's et al., study (Dahlgren et al., 2014), who found an association between decreased depressive symptoms after cognitive remediation, and an improvement in the BRIEF‐P ‘shift’ scale in adolescents with AN. Similarly, Giel et al. (2012) showed a moderate significant association between depressive symptoms and set‐shifting in adults with AN, as assessed by the WCST, the TMT and a go/no‐go task. Finally, Billingsley‐Marshall et al. (2013) also report an association between state anxiety and set‐shifting in adults with AN, as assessed by verbal fluency. Concerning ASD our results are in agreement with Hollocks' et al., study (Hollocks et al., 2014), who found a strong association between anxiety and set‐shifting, as evaluated by a card sorting task and the TMT in adolescents with ASD. Presents findings contrast however with others suggesting no influence of anxiety and depression on set‐shifting in adults with AN, as measured by different neuropsychological tasks such as the WCST and the TMT (Roberts et al., 2010; Van Autreve et al., 2013). It should be noted that some studies measure anxiety and/or depression with distinct assessing scales, also preventing further comparisons between studies. That is the case of Roberts's et al., study (Roberts et al., 2010), who used the hospital anxiety and depression scale, commonly used for screening anxiety and depressive symptoms in the non‐psychiatric populations, as opposed to the STAI and the CDI. In addition, it is possible that the unstructured nature of the BRIEF when assessing EF in everyday life, may be more sensitive to the influence of factors such as depression and anxiety, for example, via abulia, which can significantly alter the daily behaviours it assesses. However, the diagnosis of ASD remained, in our study, the variable with the most weight after adjustment for anxiety and depression.

Finally, in AN, our study finds an association between BMI with executive performance, particularly in the MI. This contradicts Calderoni's et al. study (Calderoni et al., 2013), who found no influence of BMI on EF as assessed by the NEPSY‐II in adolescents with AN, as well with as Galimberti et al. (2013), in their study with adults with AN where EF were assessed with the WCST. However, these studies included much smaller samples sizes, including only 33 and 29 patients with AN, respectively. Our results are nevertheless in agreement with Herbrich et al. (2018) who found an association between lower BMI and poorer performance at the TMT in a sample of 101 adolescents with AN, and with Lozano‐Serra's et al., study (Lozano‐Serra et al., 2014) who reported an improvement in EF with the WCST and TMT after 6 months of treatment including renutrition of adolescent girls. Although it is clinically recognized that renutrition improves cognitive functions, the literature remains in this regard too inconsistent. Moreover, our results showed a preponderance of variation in autistic traits, assessed by the AQ, with alterations in the BRI and in the GEC. This finding is reminiscent with those of Westwood et al. (2017), who found more flexibility deficits on the WCST in 99 adolescents and adults with AN presenting more autistic traits, as assessed by the ADOS‐2. Indeed, few studies have investigated the influence of autistic traits on patients with AN in relation to executive functioning, and it would be of great interest to study this parameter more systematically to complete account to these findings.

Overall, the findings of the present study show different executive profiles in AN and ASD, but also highlight the importance of autistic features in the alteration of EF, whether they are considered at a broader diagnostic level for the whole sample, or as autistic traits in adolescents with AN.

Certain limitations should be however considered. Firstly, we did not include a control group, but the structure of the BRIEF allows a comparison to a normative sample. Indeed, other studies have used these population norms to compare BRIEF scores with samples including patients with AN and ASD (e.g., Timko et al., 2021). This same BRIEF structure did not allow us to conduct logistic regression analyses on the potential association between age and sex with EF, as the dependent variable (*T*‐score) already accounted for age and sex. Although our groups differed greatly regarding the sex ratio of ASD and AN. However only one study reports sex differences in EF in ASD (White et al., 2017), with females adolescents presenting greater EF difficulties than males, as assessed by the BRIEF‐P. As it remains a question with no definite answer, we cannot affirm that this difference compromises our findings. Second, our study included a small ANBP sample, from which one could suspect of a type 1 error bias. This is however, one of the largest samples among similar studies found in the literature. Finally, while the BRIEF‐P has advantages, it also has its shortcomings. As a questionnaire, the BRIEF remains sensitive to biases of the informant observations (conflicts, psychological exhaustion, etc.). Moreover, the BRIEF does not directly assesses executive functions but rather, it assesses actual behavioural manifestation of executive function or dysfunction in everyday life. Indeed the absence of a significant correlation between the BRIEF and neuropsychological tests, highlights the interest of using the BRIEF when identifying disturbances in behaviour and overall functioning (Mcauley et al., 2010; Toplak et al., 2013). That is, the BRIEF allows to identify the impact of EF in the patient's daily life. We note that if the *z*‐scores of the AN group are less than 1.5, the ‘shift’ scale and the BRI are close to it. In short, the BRIEF scores seem to reflect the feeling of *self‐ineffectiveness* in daily functionality, identified in adolescent girls with AN.

In sum, to our knowledge, this is one of the first studies directly comparing patients with ASD and AN in terms of executive functioning on such a large sample. Our findings show distinct executive profiles for these two groups, with significantly greater difficulties for the ASD group. On the other hand, a closer analyses suggests that patients with AN‐BP seem to present more difficulties than the patients with AN‐R group. The present findings show an association between anxiety and depression, and the EF of the participants, but also a preponderant connection between variations in EF and the diagnosis of ASD. It is interesting to note, that beyond diagnosis, there is a significant association between the autistic traits assessed by the AQ and EF in patients with AN. However, further studies are needed to confirm these findings and to better characterise eating‐related symptomatology in individuals with ASD relative to individuals with AN. It would also be of great interest to deepen our knowledge regarding of the different AN subtypes in which EF are altered. A fine grained analyses and comprehension of EF in the different AN subtypes, in relation with the presence, or not, of autistic traits may open new avenues to better explain the discrepancies found in the literature, but most importantly, it may help targeting which patients may benefit the most from EF‐oriented treatment interventions. This would allow developing more specific and adapted interventions in the form of cognitive remediation programs, known to successfully aid individuals with ASD.

## CONFLICTS OF INTEREST

The authors declare no conflicts of interest.

## CONSENT TO PARTICIPATE

Participants and/or parental informed consent were obtained from all participants at the time of testing.

## CONSENT FOR PUBLICATION

All authors consent to terms of publication.

## Data Availability

The data that support the findings of this study are available from the corresponding author upon reasonable request.
